# Spatial-Temporal Attention Mechanism and Graph Convolutional Networks for Destination Prediction

**DOI:** 10.3389/fnbot.2022.925210

**Published:** 2022-07-06

**Authors:** Cong Li, Huyin Zhang, Zengkai Wang, Yonghao Wu, Fei Yang

**Affiliations:** ^1^School of Computer Science, Wuhan University, Wuhan, China; ^2^Department of Information Engineering, Wuhan Institute of City, Wuhan, China; ^3^College of Information Science and Engineering, Jiaxing University, Jiaxing, China

**Keywords:** destination prediction, graph convolution network (GCN), attention mechanism, spatial-temporal correlation, long-short term memory network (LSTM)

## Abstract

Urban transportation destination prediction is a crucial issue in the area of intelligent transportation, such as urban traffic planning and traffic congestion control. The spatial structure of the road network has high nonlinearity and complexity, and also, the traffic flow is dynamic due to the continuous changing of the traffic environment. Thus, it is very important to model the spatial relation and temporal dependence simultaneously to simulate the true traffic conditions. Most of the existing destination prediction methods have limited ability to model large-scale spatial data that changes dynamically with time, so they cannot obtain satisfactory prediction results. This paper proposes a human-in-loop Spatial-Temporal Attention Mechanism with Graph Convolutional Network (STAGCN) model to explore the spatial-temporal dependencies for destination prediction. The main contributions of this study are as follows. First, the traffic network is represented as a graph network by grid region dividing, then the spatial-temporal correlations of the traffic network can be learned by convolution operations in time on the graph network. Second, the attention mechanism is exploited for the analysis of features with loop periodicity and enhancing the features of key nodes in the grid. Finally, the spatial and temporal features are combined as the input of the Long-Short Term Memory network (LSTM) to further capture the spatial-temporal dependences of the traffic data to reach more accurate results. Extensive experiments conducted on the large scale urban real dataset show that the proposed STAGCN model has achieved better performance in urban car-hailing destination prediction compared with the traditional baseline models.

## 1. Introduction

According to the statistics of the China Online car-hailing Regulatory Information Exchange Platform, as of 31 July 2021, the platform received 7,76,564 million orders in July, up 10.7% from the previous month. With the increasing demand for online car-hailing services, online car-hailing has become an important choice for residents' daily travel. A large amount of online car-hailing data is generated during daily travel, these data include valuable information such as the user's travel time, departure place, and destination (Hu et al., 2021). If these data can be used to accurately predict the travel destination of online car-hailing, and estimated travel time, more flexible scheduling of online car-hailing can improve its utilization rate and reduce the empty load rate. Through destination prediction, the distribution of traffic hot spots in the future can be obtained, so that congestion areas can be predicted, timely evacuation and urban traffic operation efficiency can be improved, it will be beneficial to urban traffic management and urban planning (Liu et al., 2015; Li et al., 2021a), congestion area prediction (Kan et al., 2019; Guo et al., 2021), and urban taxi scheduling (Xu et al., 2018; Rossi et al., 2020). In addition, destination data not only indicate the user's location information, but also hides a wealth of user behavior preferences, user interests, and other information, through purpose prediction, you can also obtain nearby places that users may be interested in and send targeted advertisements based on destinations (Wang et al., 2017; Zhang et al., 2018). Finally, predicting users' trip and destination preferences based on their historical travel behavior will also play an important role in the rise of autonomous driving in recent years. Therefore, destination prediction of car-hailing is not only an important task of car-hailing platform management but also a key task of intelligent transportation.

Driven by a large number of historical order data (such as driving time, driving track, pickup point, and disembarking point) and other extended information (holidays, POI, weather conditions, etc.), travel destination prediction has always been a research hotspot. Most of the early destination prediction methods are based on probability theory, and the model is based on the Markov chain (Zong et al., 2019) or hidden Markov chain (Dantas Nobre Neto et al., 2018; Rezaie and Li, 2021) is the most widely used destination prediction method. These models divide trajectories into path segments and, as states of Markov processes, predict future destinations by calculating transition probabilities of historical trajectories. The above methods have achieved a good prediction effect through learning historical trajectory data, but the following two problems may also occur in the prediction process: (1) The historical track data set has the characteristics of space-time, complexity, and mass. If the noise of the data set is too large, the accuracy of prediction will decrease. (2) In the process of prediction, the spatial-temporal relationship between tracks is ignored, resulting in the problem of the sparsity of data, resulting in an inaccurate destination prediction.

In order to solve the problem of noise and sparsity of historical data, researchers began to apply deep learning technology to travel destination prediction. These studies include Multi-Layer Perceptron (MLP) (Song et al., 2020b), Convolutional Neural Network (CNN) (Miao et al., 2022), Recurrent Neural Network (RNN) (Fu and Lee, 2020), LSTM (Gui et al., 2021), these methods have achieved good prediction results. However, the following three problems also arise when predicting destinations: (1) Some existing deep learning methods are based on only partial information or partial and external information and do not make full use of spatial-temporal information. (2) Many existing deep learning-based methods for spatial-temporal data stitch together different features and input them into the prediction model, resulting in poor prediction results. (3) A part of the research focuses mostly on the neural network hierarchical changes and the mutual combination of each model, ignoring the features and relationships of spatial-temporal in destination prediction, resulting in the loss of spatial-temporal information.

In view of the above problems, this article proposed a spatial-temporal attention combined graph convolutional network model to solve the fusion of spatial-temporal information and predict the destination of online car-hailing. The main contributions of this article are summarized as follows:

- We propose the STAGCN model. This model predicts the number of orders at a given time period in a novel and unified way, which can significantly help online car-hailing scheduling and urban traffic management.
- We describe the problem of STAGCN by dividing urban areas into grids on the map. Then, we designed a grid embedding network to perform embedding for each grid by graph convolution between the newly defined region adjacency matrix and the relational adjacency matrix. Use GCN to capture spatial-temporal correlation.
- We have developed a spatial-temporal attention mechanism. In the destination prediction, the relative importance of each position in the time series of the day is dynamically calibrated through the weight distribution method; the space attention mechanism can identify the importance of the position sequence of each day. The relative importance of the target location is to be predicted.
- We designed a multi-task learning network to predict the destination of online car-hailing with the help of an LSTM. A large number of experiments on real and large-scale car-hailing data sets show that the proposed STAGCN model is better than the baseline.

## 2. Related Study

With the proliferation of mobile sensors and smartphone devices, people are increasingly benefiting from various location services. Online car-hailing destination prediction is one of the hot research topics in recent years. Accurate and efficient destination prediction helps to organize traffic flow, improve vehicle utilization, reduce waiting time, and ease traffic congestion. Scholars at home and abroad have conducted relevant research on online taxi destination prediction methods and have achieved some research results in this field. The relevant studies are mainly classified into the following three categories:

(1) **Traditional statistics methods**. Alzyout et al. (2019) use the improved ARIMA model to predict the future position of the vehicle through the historical positioning data of the vehicle. Wiest et al. (2012) use a Gaussian mixture model to predict vehicle travel destinations. This method builds a Gaussian mixture model, then learns the probability distribution between travel trajectories and travel destinations from historical travel data, and then predicts travel destinations by calculating the conditional probability distribution of travel destinations relative to the current travel trajectory. However, these methods only take into account the effect of the current location on future destinations, resulting in less accurate predictions. Besse et al. (2018) use a clustering approach to cluster historical travel trajectories into a certain number of categories by similarity and then calculate the category to which the current trajectory belongs and uses the average of all travel destinations in that category as the predicted travel destination. However, the accuracy of the prediction is affected when the number of historical location points is large. Lassoued et al. (2017) perform spatial clustering of vehicle historical trajectories to predict future destinations. However, the global-based trajectory clustering ignores the detailed information of sub-trajectories, which affects the accuracy of prediction.

(2) **Classical machine learning methods**. In recent years, researchers have tried to solve this problem using machine learning methods. Zong et al. (2019) used a Hidden Markov Model (HMM) for training to set the initial state and parameters to obtain the hidden state of the GPS data sequence, which is the real-time endpoint, based on the previously counted visit frequency and support points of different regions. According to the estimation results of the HMM, the frequently visited destinations have the highest transfer probability of being identified as the next destination. Gambs et al. (2012) proposed a Mobile Markov Chain (MMC) mobility model is proposed to predict the next location of an individual based on observations of his mobility behavior over time and the locations he has visited recently. The mobility behavior of an individual is modeled as a discrete stochastic process in which the probability of moving to a state depends only on the previously visited state and the probability distribution of transitions between states. The above methods can effectively realize the destination prediction by learning the historical trajectory. However, the prediction method of the Markov depends on the historical data, does not consider the dependence of spatial-temporal, and ignores the impact of the sparseness of the data in the prediction process. Lv et al. (2020) modeled the historical trajectory as the data of graph structure without time information, used the CNN model to learn the hierarchical features of the graph, and embedded the graph information into the model prediction unit to predict the destination. Cui et al. (2020) proposed the TGC-LSTM model to represent the traffic road network as a graph structured network to learn deep correlation features and use LSTM to predict the traffic state of the whole spatial network. The above neural network learning methods for destination prediction ignore the important feature of spatial-temporal correlation, and the need for iterative training will lead to a gradual accumulation of errors.

(3) **Deep learning methods**. The key to solving the problem of destination prediction is to make full use of the deep features of traffic spatial-temporal data. Many studies have begun to improve the graph convolution network model to transform complex traffic data into Euclidean spatial graph and capture spatial features between vertices of grid graph with spatial information. Yu et al. (2018) proposed a Spatial-Temporal Graph Convolution Network (STGCN) model to transform the prediction problem of nonlinear structure into the prediction problem of time series. The STGCN model improves the conventional recursive module and convolution unit in the traditional model. The GCN module captures the spatial-temporal characteristics of the traffic graph and predicts the traffic flow through the time series model. Song et al. (2020a) proposed an STSGCN model for traffic congestion prediction. In this model, a Spatial-Temporal Synchronization Mechanism is designed through the GCN network to solve the problem of spatial-temporal asynchronism in the traffic network diagram. STSGCN can effectively capture the complex temporal and spatial correlation between roads in the traffic map and predict traffic conditions. The above GCN model relies on the eigenvalues of the Laplacian matrix, which makes it difficult to extract convolution operations from the entire static graph structure. Therefore, the destination prediction problem relies on well-defined graph structure information to efficiently extract spatial-temporal features and model them more finely.

The research shows that the destination of online car-hailing has certain periodic regularity (Zhang et al., 2020). Many studies have introduced attention mechanisms into destination prediction tasks. Guo et al. (2019) proposed a multi-component ASTGCN model based on a time segment attention module to solve the problems of spatial-temporal modeling and traffic prediction. Do et al. (2019) introduced the attention mechanism into traffic flow prediction and proposed the STANN model. Spatial attention focuses on the dependence between roads in the graph structure, and temporal attention captures the time dependence between time steps in the spatial-temporal graph. The weight of spatial and temporal attention was obtained by the GRU, and the final prediction result was obtained. The core of the attention mechanism is to assign weight to a given piece of information, and a high weight means that the model needs to focus on it. Therefore, an important issue to be addressed in the above research is how to assign the weights of attention.

According to the above research, the destination location of car-hailing has the characteristics of periodicity, correlation, and dependence. Therefore, the core of destination prediction lies in how to capture the high-dimensional features in the historical data of car-hailing, especially in the time and space dimensions. In this article, GCN is used to extract spatial dimension features from graph structure data. The historical orders are divided into fine granularity in a periodic, and the convolution operation is carried out on the time axis to obtain the characteristics of the time dimension. The attention mechanism is adopted to assign different weights to features with strong periodicity and high correlation, thus effectively solving the problem of destination prediction of car-hailing.

## 3. Proposed Method

### 3.1. Description of the Problem

In this article, we need to solve a data set prediction problem including spatial hierarchy and time series structure, so we transform the spatial-temporal data represented by grid matrix into a spatial-temporal fusion graph. Considering the goal-orientation of mobile travel behavior, regular grids with equal spatial scale are used to divide spatial regions discretely, and the destination order data of car-hailing is mapped to the corresponding spatial grids, and then the spatial sequence data represented by the transformed grid ID numbers are analyzed and mined for spatial dependence. Online car-hailing destination order data has a clear time correlation, so the short-term sequence of adjacent time slots is combined with the long-term sequence of several days or weeks in the near future to more accurately model the time dependence of the data. This chapter expands the definition and description of the destination prediction model through the definition of the problem description, spatial grid, destination area, and time slot destination matrix.

**Definition 1 Problem description**. Suppose this article uses a dynamic observation system on a spatial region represented by *M* × *N* grids composed of *M* rows and *N* columns. In each cell in the grid, there are *P* measurement values that change with time Variety. Therefore, observations at any time can be expressed in terms of *X* ∈ *R*^*P*×*M*×*N*^, Where *R* represents the observed characteristic domain. The spatial-temporal series prediction problem is converted to historical *i* observations to predict the future *t* + 1 time series $\left\{\hat{x_{1}},\hat{x_{2}},...,\hat{x_{t}}\right\}$. The detailed equation is shown in Equation (1).

(1) $$\begin{array}{l} {\hat{X}}_{t+1}=\underset{{\hat{x}}_{t+1}}{\text{arg max }\rho }\left({\hat{X}}_{t+1}|X_{t-i+1},\cdots \;,X_{t}\right) \end{array}$$

**Definition 2 Spatial grid**. In this article, we adopt the classical treatment in the field of transportation (Liu et al., 2019), dividing the area to be treated equally into several grids, each defined by the maximum and minimum coordinates (Mahajan et al., 2022). In this way, the number of destination orders of the online car-hailing in each small grid area is studied. Subsequently, each grid is considered as a vertex of the graph, which is used to construct a graph model for the prediction of online taxi destinations. We define the spatial grid as an undirected graph *G* = (*V, E, A*), where *V* is the set of nodes, ∣*V*∣ = *N* means there are *N* nodes in the graph; use the edges of a graph to represent the connectivity of nodes in the graph, *E* is the set of edges, *A* ∈ ℝ^*N* × *N*^ is the spatial adjacency matrix representing the distance dependence between nodes. Each node on the spatial grid *G* will detect *P* time series data with the same sampling frequency, i.e., each node will generate a feature vector of length *P* at each time stamp (Li et al., 2021b).

**Definition 3 Destination grid**. Due to the influence of the workday cycle, morning and evening peak cycle, People's Daily work and rest patterns, and other factors, the destination area often has a strong similarity at the same time on weekdays or weekends. We divide the traffic network into *M* × *N* grids, and each grid represents a destination node. Consider the accumulated drop-off points in a certain time interval t (Ma et al., 2021). The destination of the passenger in the time interval t of the grid located in the *i*-th row and *j*-th column is defined as Equation (2).

(2) $$\begin{array}{l} x_{t}^{Des,i,j}=\underset{T_{r}\in T_{s}}{\sum }\{i>1|d_{i}\in \left(i,j\right)\wedge d_{\left(i+1\right)}\notin \left(i,j\right)\} \end{array}$$

**Definition 4 Destination volume matrix**. The purpose of our study is to predict the volume of destination orders in the future based on the history data. Thus, we establish the destination volume matrix for the historical data. Given the destination grid indexed (*i, j*) and the time slot indexed t, we define the entry $O_{i,j}^{t}$ as the volume of destination orders in such spatial-temporal background. Specifically, the destination volume matrices are established every 60 min. The detailed equation is shown in Equation (3).

(3) $$\begin{array}{l} D_{t}=\left(\begin{array}{cccc} O_{1,1} & O_{1,2} & \cdots & O_{1,n}\\ O_{2,1} & O_{2,2} & \cdots & O_{2,n}\\ \vdots & \vdots & \cdots & \vdots \\ O_{m,1} & O_{m,2} & \cdots & O_{m,n} \end{array}\right)\; \end{array}$$

### 3.2. The Framework of the Proposed Method

**Figure 1:**
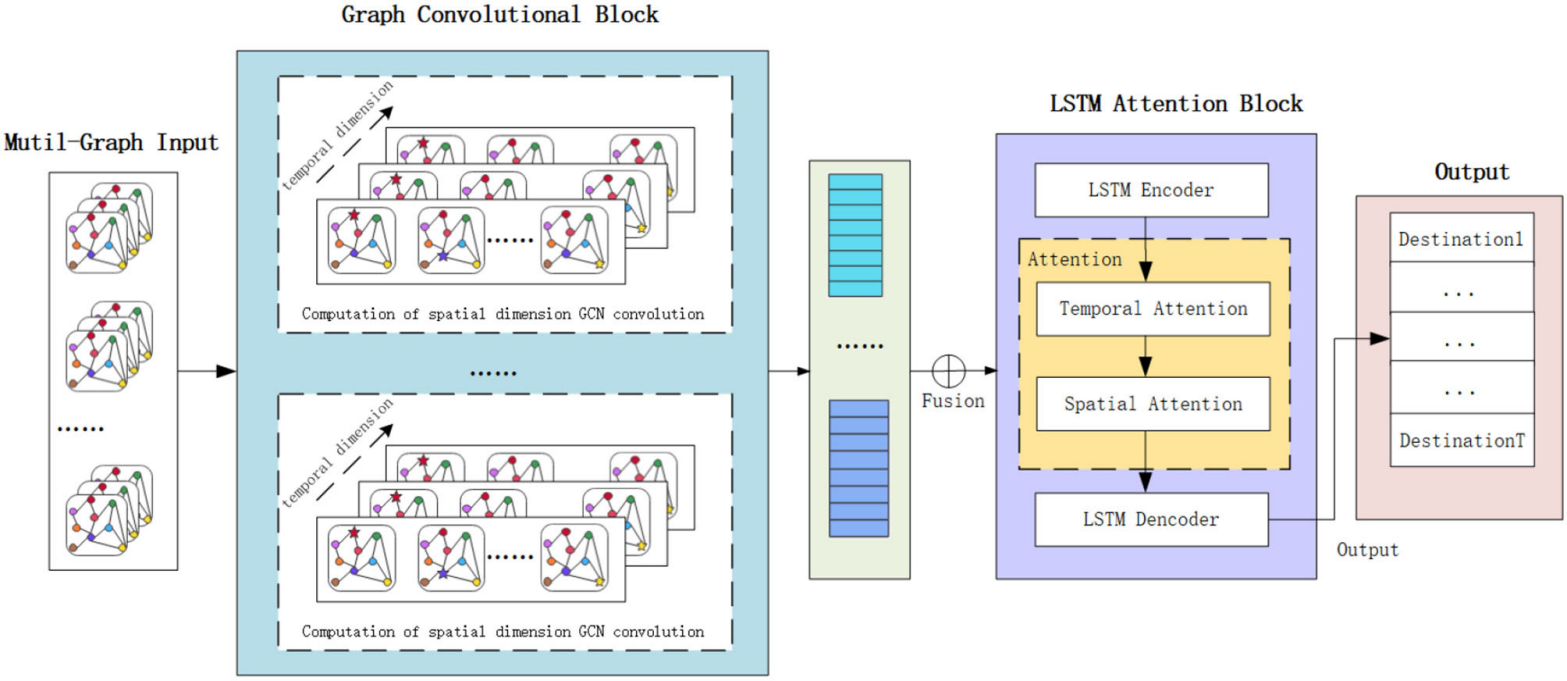
The framework of the proposed Spatial-Temporal Attention and Graph Convolutional Network Model (STAGCN).

The framework of the proposed Spatial-Temporal Attention and Graph Convolutional Network Model (STAGCN) is shown in Figure 1. The GCN model with two hidden layers is utilized to learn the latent low dimensional representations of the multi-graph input data. The output of the GCN is time series data, which is fed into the LSTM encoder model to capture the temporal dependence inside the time series data. The hidden output of the LSTM encoder in the different time slots is fed into the attention module to learn the spatial and temporal weight vectors. Finally, the weighted spatial-temporal vectors are used as the input of the LSTM decoder to predict the volume of destination orders in the future. The LSTM encoding module is mainly used to model the temporal relationship between location sequences, and the decoding module makes predictions for future location sequences. For example, from the correlation between spatial location and the surrounding area, the surrounding area has a strong or weak impact on the prediction of the destination, from the time working day, non-working day periodicity on the prediction of the destination? The impact of different time data on the current forecast? Prediction from LSTM alone is not sufficient for forecasting. Adding spatial-temporal attention mechanism gives different weights from spatial location and time to effectively capture temporal and spatial dimensional features and their spatial-temporal correlation.

### 3.3. Input Construction

The use of grid matrices to represent graph convolutional neural networks have been applied in many previous studies (Jin et al., 2020). The data model of the grid matrix can better represent non-Euclidean structural features and capture local spatial features. For example, certain destination areas are at the center of the transportation network of a city and have strong connections with other destinations, which represents a stronger correlation between areas. Partitioning the grid matrix by time gaps and arranging it into multiple grid matrices establishes correlations between spatial and temporal dynamics. We divide map areas into uniform spatial grids by latitude and longitude, and each grid is identified by a unique identifier. Grid identifiers are assigned linearly, ranging from 1 to the number of grids. Therefore, the destination sequence of online car-hailing is represented as a grid identifier vector in a spatial graph. However, the grid identifier vector cannot represent the spatial-temporal dependence between grids, and cannot be directly applied to graph convolutional networks due to their data types. In order to capture the dependency between grids and make grids suitable for graph convolutional networks, we use an embedding method to transform each grid into a set of real-valued vectors with spatial dependence, and embed spatial distance values into spatial grid matrixes (Li et al., 2020).

**Region adjacency matrix**: By analyzing the historical order data of online car-hailing, the two adjacent regional grids have the characteristics of a strong correlation. According to this characteristic, we construct the adjacency matrix with the distance between adjacent regions. We use the reciprocal of the center distance to mark the dependency weight between the two regions. In this way, the closer the distance, the higher the weight value and the stronger the dependence (Jin et al., 2020). The weight equation is shown in Equation (4), the region adjacency matrix is shown in Equation (5).

(4) $$\begin{array}{l} G_{d}\left(V,E\right),Weight=\{\begin{array}{l} d^{-1},i\neq j\;d\leq \delta \\ 1,i=j\\ 0,i\neq j\;d>\delta \end{array} \end{array}$$

(5) $$\begin{array}{l} a_{t}^{d}=\left(\begin{array}{cccc} O_{1,1} & O_{1,2} & \cdots & O_{1,n}\\ O_{2,1} & O_{2,2} & \cdots & O_{2,n}\\ \vdots & \vdots & \cdots & \vdots \\ O_{m,1} & O_{m,2} & \cdots & O_{m,n} \end{array}\right) \end{array}$$

**Destination relation matrix**: Construct a spatial correlation graph of car-hailing destination counts. By calculating the destination count for each time period and each area, and then calculating the correlation between every two areas as the link weight in the graph. We use the historical order volume of online car-hailing to construct the relationship matrix between destinations. The historical drop-off of each grid in each time slot (60 min) was counted, and then the correlation between every two grids was calculated as the weight of the destination relationship matrix (Liu et al., 2019). Pearson coefficient is a measure of the degree of linear correlation between two variables, which is widely used to measure the spatial linear correlation. When the distance between regions deviates greatly from the average level, the distance cannot well reveal the real similarity. In this case, the Pearson coefficient is used to calculate the destination spatial correlation (Shan et al., 2014; Faroqi et al., 2017), as shown in Equation (6). The weight equation is shown in Equation (7), When *r* < 0.05, we believe that there is no significant spatial correlation between the two grids, so it is defined as 0. *r*_*i*_*j* represents the Pearson correlation between grid *i* and grid *j*, destination relationship is shown in Equation (8).

(6) $$\begin{array}{l} r=\frac{\overset{99}{\underset{i=1}{\sum }}\left(x_{i}-\bar{x}\right)\left(y_{i}-\bar{y}\right)}{\sqrt{\overset{99}{\underset{i=1}{\sum }}{\left(x_{i}-\bar{x}\right)}^{2}}\sqrt{\overset{99}{\underset{i=1}{\sum }}{\left(j_{i}-\bar{j}\right)}^{2}}} \end{array}$$

(7) $$\begin{array}{l} G_{d}\left(V,E\right),Weight=\{\begin{array}{c} r,\;r\geq 0.05\\ 0,\;r<0.05 \end{array} \end{array}$$

(8) $$\begin{array}{l} a_{t}^{r}=\left(\begin{array}{ccccc} 0 & r_{0,1} & \cdots & r_{0,j-2} & r_{0,j-1}\\ r_{1,0} & 0 & \cdots & r_{1,j-2} & r_{1,j-1}\\ \vdots & \vdots & \ddots & \vdots & \vdots \\ r_{i-2,0} & r_{i-2,1} & \cdots & 0 & r_{i-2,j-1}\\ r_{i-1,0} & r_{i-1,1} & \cdots & r_{i-1,j-2} & 0 \end{array}\right) \end{array}$$

**Graph fusion**: The destination volume graph, region adjacency graph, and destination relation graph are fused into a fusion graph with multi-graph characteristics. We perform fusion by weighting the adjacency matrix of the above graph. Since the adjacency matrix values of different graphs may differ greatly, we first normalize the adjacency matrix *A* of each graph above.

(9) $$\begin{array}{l} A^{\prime }=D^{-1}A+I \end{array}$$

where D is:

(10) $$\begin{array}{l} D=\left(\begin{array}{cccc} \sum_{j=0}^{n-1}A_{0,j} & 0 & \ldots & 0\\ 0 & \sum_{j=0}^{n-1}A_{1,j} & \ldots & 0\\ \vdots & \vdots & \ddots & \vdots \\ 0 & 0 & \ldots & \sum_{j=0}^{n-1}A_{n-1,j} \end{array}\right) \end{array}$$

The results of the above weighted sum operation are further normalized and the softmax operation is carried out in the weighted matrix. Now the above three figures are fused, and the fusion formula is as follows:

(11) $$\begin{array}{l} \begin{array}{c} W_{i}^{\prime }=\mathrm{Softmax}\left(W_{i}\right)\\ A_{i}^{\prime }=D_{i}^{-1}A_{i}+I\\ F=\overset{N}{\underset{i=1}{\sum }}W_{i}^{\prime }◦A_{i}^{\prime }\;\left(1\leq i\leq 3\right) \end{array} \end{array}$$

where ◦ is the Hadamard product operation, and *F* is the graph fusion result that will be used in the graph convolution network module.

### 3.4. Graph Convolution Networks

Graph convolutional networks have achieved great success in dealing with spatial relations in non-Euclidean Spaces. In this article, the graph convolution network theory proposed by Kipf and Welling (2017) is used to construct a spatial graph convolution module to capture spatial-temporal correlation. The graph convolution module consists of a set of graph convolution operations, which can aggregate the characteristics of each node and its neighbors. Graph convolution is used in the vertex domain to obtain the dependency of the spatial neighborhood in destination grids. The input of graph convolution is the graph vector matrix of the destination grids. In the graph convolution operation, each node aggregates the characteristics of itself and its neighbors at adjacent time steps. The aggregation function is defined by designing the attention mechanism. Different from the correlation model mentioned in Chapter 2, the attention mechanism pays more attention to the dependence between nodes in the adjacency matrix. We deploy a fully connected layer with an activation function to transform the characteristics of the node into a new space. The graph convolution operation is shown in Equations (12, 13).

(12) $$\begin{array}{l} H^{l+1}=f\left(H^{l+1},A\right)=\sigma \left(D^{-\frac{1}{2}}AD^{-\frac{1}{2}}H^{l}W^{l}\right) \end{array}$$

(13) $$\begin{array}{l} H^{l^{\prime }}={\hat{D}}^{-\frac{1}{2}}A{\hat{D}}^{-\frac{1}{2}}H^{l} \end{array}$$

where *I* is the identity matrix, $\hat{A}=A+I$, *A* ∈ *R*^(*n* × *n*)^ is expressed as the adjacency matrix, the diagonal node degree matrix of $\hat{A}$ is expressed as $\hat{D}$, the weight of the *l*-th layer matrix is represented as *W*^*l*^, *H*^*l*^ represented the feature of the *l*-th layer, for the input layer, *H*^0^ is *X*, *H*^*l*′^ ∈ *R*^(*n* × *m*)^ is a feature matrix with local spatial structure, and σ(·) is a nonlinear activation function.

According to the principle of GCN, the convolution of the graph can be expressed as follows by the above formula:

(14) $$\begin{array}{l} h_{t^{\prime }}^{0}=F*x_{t}^{\prime }\;t^{\prime }\in \left[0,t-1\right] \end{array}$$

(15) $$\begin{array}{l} h_{t}^{\prime 1}=\left(h_{t^{\prime }}^{0},a_{t}\right)=\sigma \left({\left({\hat{D}}^{-\frac{1}{2}}A{\hat{D}}^{-\frac{1}{2}}\right)}^{1}h_{t^{\prime }}^{0},W^{\left(1\right)}\right) \end{array}$$

(16) $$\begin{array}{l} h_{t}^{\prime 2}=\left(h_{t^{\prime }}^{1},a_{t}\right)=\sigma \left({\left({\hat{D}}^{-\frac{1}{2}}A{\hat{D}}^{-\frac{1}{2}}\right)}^{2}h_{t^{\prime }}^{1},W^{\left(2\right)}\right)\\ \ldots \ldots \end{array}$$

(17) $$\begin{array}{l} h_{t}^{\prime n}=\left(h_{t^{\prime }}^{n-1},a_{t}\right)=\sigma \left({\left({\hat{D}}^{-\frac{1}{2}}A{\hat{D}}^{-\frac{1}{2}}\right)}^{n}h_{t^{\prime }}^{n-1},W^{\left(n\right)}\right) \end{array}$$

where $h_{t^{\prime }}^{0}$ is the initial input value and represents the spatial characteristic matrix of time slot *t*. $h_{t^{\prime }}^{1},\cdots h_{t^{\prime }}^{n}$ respectively represent the potential information in the *n* hidden layer. STAGCN model adopts two hidden layer GCN models, 20 convolution kernels are used for spatial dimension graph convolution, and 5 convolution kernels of the same size are used for temporal dimension graph convolution. The result dimension of spatial-temporal graph convolution is consistent with the target dimension by full connection operation. A fully connected module maps all the hidden node features to a 1 × 10 vector of the second hidden layer of GCN.

### 3.5. LSTM Module

**Figure 2:**
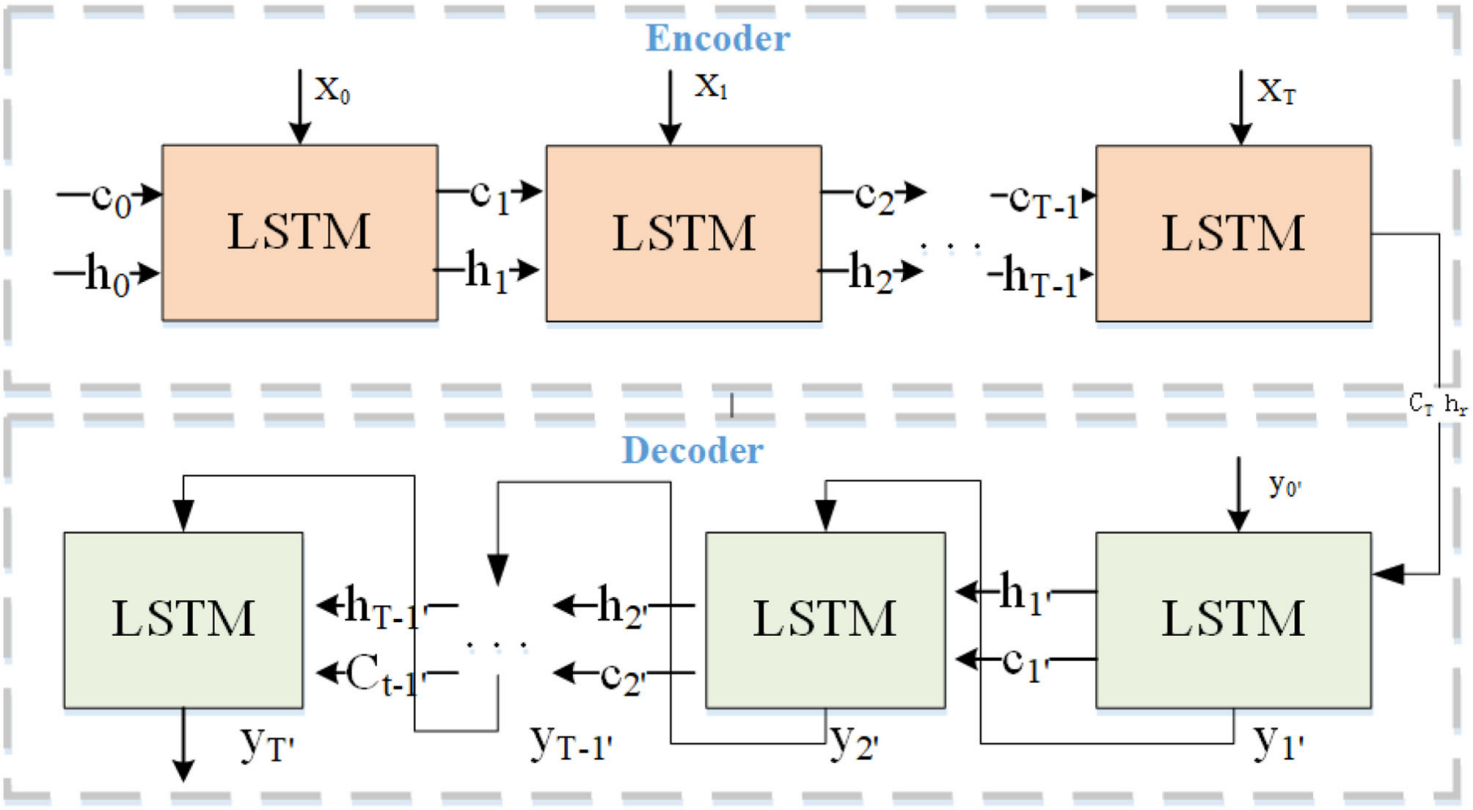
Long-Short Term Memory (LSTM) encoder-decoder framework.

Long-Short Term Memory is a type of temporal recurrent neural network that determines the storage and discarding of information by adding gating (input gates, forgetting gates, and output gates) to RNN. LSTM solves the problem of gradient dispersion of RNN models and can better portray data with spatial-temporal correlation. The LSTM prediction model based on the structure of the spatial-temporal attention encoder-decoder includes three parts: LSTM encoding network, LSTM decoding network, and spatial-temporal attention layer, as shown in Figure 2. The LSTM encoder obtains the spatial-temporal features of the destination sequence from the graph convolutional layer and constructs a deep latent spatial-temporal representation of the historical destination sequence data through the spatial-temporal attention vector. The LSTM decoder decodes the encoded spatial-temporal attention vector to predict the destination sequence.

The LSTM model has proved to be very effective in processing time-series data with long time dependent features. The LSTM encoder in this model learns its periodic time-dependence and spatial-temporal correlation of learning the long-term spatial-temporal sequence of the input, then the generated vector is combined with the spatial-temporal attention vector to construct the deep spatial-temporal feature representation of historical destination sequence data. The LSTM decoder decodes based on the encoded spatial-temporal attention vector and takes the reconstructed destination sequence as the target predictive value as shown in Figure 2. Its working mechanism is following equations (Li et al., 2020).

(18) $$\begin{array}{l} \begin{array}{l} f_{t}=\sigma \left(W_{xf}x_{t}+W_{hf}h_{t-1}+b_{f}\right)\\ i_{t}=\sigma \left(W_{xi}x_{t}+W_{hi}h_{t-1}+b_{i}\right)\\ o_{t}=\sigma \left(W_{xo}x_{t}+W_{ho}h_{t-1}+b_{o}\right)\\ c_{t}=f_{t}⊙C_{t-1}+i_{t}⊙\tanh\left(W_{xc}x_{t}+W_{hc}h_{t-1}+b_{c}\right)\\ h_{t}=o_{t}⊙\tanh\left(c_{t}\right) \end{array} \end{array}$$

where *f*_*t*_, *i*_*t*_, and *o*_*t*_ respectively forgotten gate, update gate, and output gate. *c*_*t*_ and *h*_*t*_ are cell memory state vectors and hidden state vectors, respectively. In these equations, σ is the sigmoid function, ⊙ is element wise product, and *x*_*t*_ is the input vector. *W* and *b* are the weight and bias in the training process. We simplify the LSTM representations in Equation (18) as the in Equation (19) shows.

(19) $$\begin{array}{l} h_{t},c_{t}=LSTM\left(x_{t},h_{t-1},c_{t-1}\right) \end{array}$$

The architecture consists of two models: a model to read the input sequence and encode it as a fixed-length vector, and a second model to decode the fixed-length vector and output the predicted sequence. The encoder accepts the input time series data as well as the hidden state of the previous time slot. The outputs *h*_*t*_ and *c*_*t*_ are the hidden state of the *t*-th time step and the encoder latent vector, respectively through (Equation 19).

The decoder parses the state vectors and feeds the prediction results back to the network recursively to slow down the rapid accumulation of errors with the prediction compensation and achieve a more accurate multi-step prediction. The decoder process decodes the hidden state vectors and cell state vectors generated by the encoder process to obtain the output sequence. The decoder accepts *c*_*t*_ and *h*_*t*_ passed from the encoder. First, the decoder receives *y*_0_ and uses initial vectors $y_{0^{\prime }}$, and $c_{0^{\prime }}$ to calculate $c_{1^{\prime }}$ and $y_{1^{\prime }}$ through (Equations 19, 20). $y_{1^{\prime }}$ represents the destination where the online car-hailing will go. The sequence of prediction results $y_{1^{\prime }}$ ⋯  $y_{T^{\prime }}$ is calculated through (Equation 20).

(20) $$\begin{array}{l} \begin{array}{c} Y_{t^{\prime }}=W_{l}h_{t^{\prime }}\\ y_{t^{\prime }}=\frac{e^{y_{t^{\prime }}}}{\sum_{j=1}^{t}e^{y_{j}}}\\ y_{t^{\prime }}=max\left(y_{t^{\prime }}\right) \end{array} \end{array}$$

### 3.6. Spatial-Temporal Attention Mechanism

**Figure 3:**
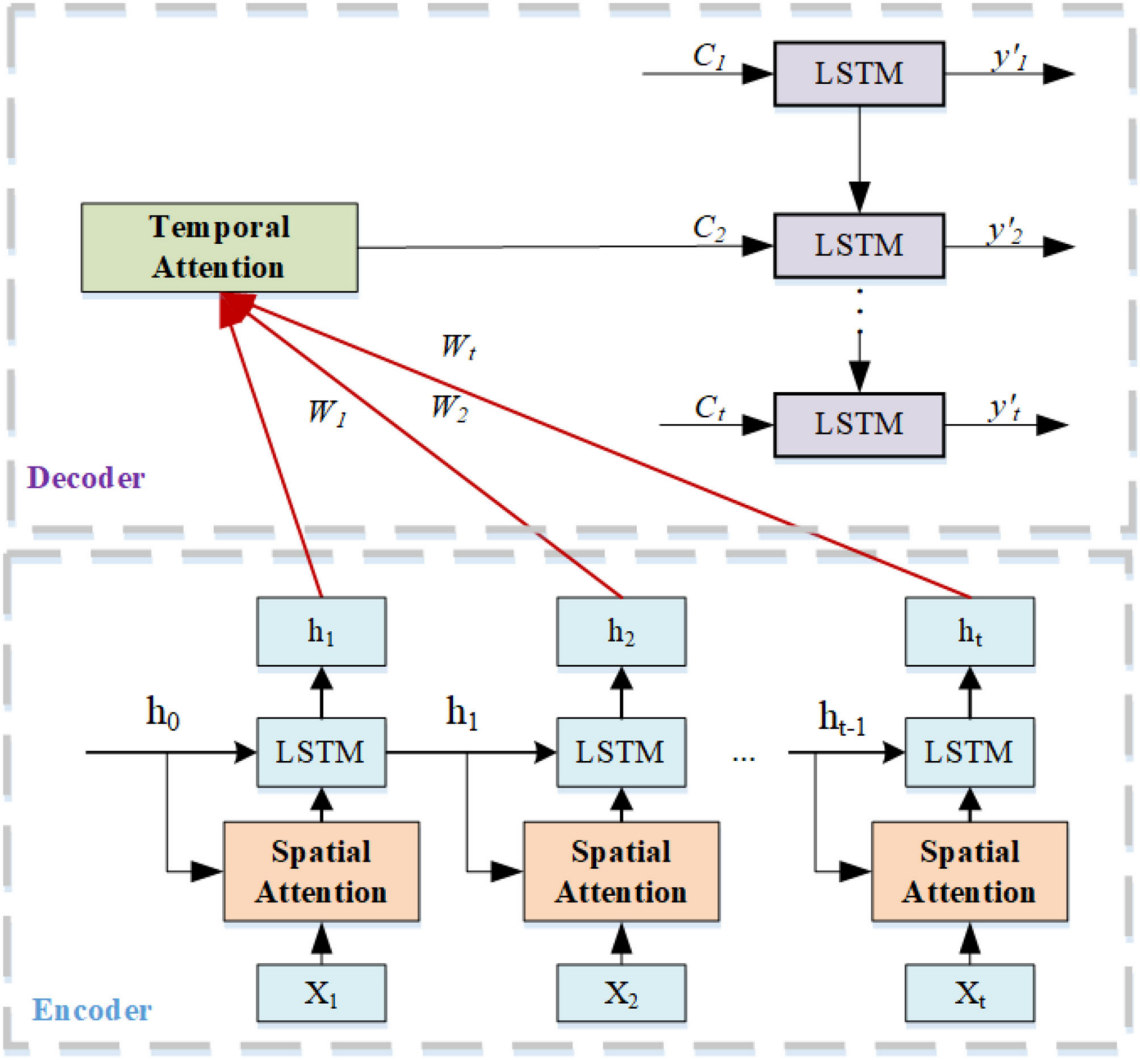
Spatial-temporal attention mechanism.

We use the spatial-temporal attention mechanism to capture the dynamic spatial-temporal correlation on the transportation network (Figure 3). The temporal attention mechanism can dynamically calibrate the relative importance of each position in the time series of the day through weight distribution; the spatial attention mechanism can identify the relative importance of the position sequence of each day to the target position to be predicted.

#### 3.6.1. Spatial Attention Mechanism

In terms of spatial dimension, the location of passengers arriving at their destination influences each other, which is highly dynamic. Therefore, we use attentional mechanisms to adaptively capture dynamic associations between nodes in spatial dimensions (Guo et al., 2019). Here, we use the attentional mechanism to adaptively capture dynamic correlations between nodes in spatial dimensions. The calculation method of the spatial attention mechanism is shown in Equations (21, 22).

(21) $$\begin{array}{l} S=V_{s}\cdot \sigma \left(\left(x_{i}^{\left(r-1\right)}W_{1}\right)W_{2}{\left(W_{3}x_{i}^{\left(r-1\right)}\right)}^{T}+b_{s}\right) \end{array}$$

(22) $$\begin{array}{l} S_{i,j}^{\prime }=\frac{\exp\left(S_{i,j}\right)}{\sum_{j=1}^{N}\exp\left(S_{i,j}\right)} \end{array}$$

where $x_{i}^{\left(r-1\right)}$ is the input of the *r*-th spatial-temporal block. *C*_*r*−1_ is the number of channels of the input data in the *r*-th layer. *V*_*s*_,*b*_*s*_ ∈ℝ^ℕ × ℕ^, $W_{1}\in {\mathbb{R}}^{T_{r-1}}$, $W_{2}\in {\mathbb{R}}^{C_{r-1}\times T_{r-1}}$, $W_{3}\in {\mathbb{R}}^{C_{r-1}}$ are learnable parameters, and sigmoid σ is used as the activation function. The spatial attention matrix *S* is dynamically computed according to the current input of this layer. The value of an element *S*_*i,j*_ in *S* semantically represents the correlation strength between node *i* and node *j*.

#### 3.6.2. Temporal Attention Mechanism

The temporal attention mechanism analyses the temporal behavior features vector by capturing the encoder's temporal hiding state during future temporal vector generation (Guo et al., 2019; Tao et al., 2020). The temporal attention mechanism is essentially the weighted sum of the sequence $\left\{x_{t^{\prime }},1\leq t^{\prime }\leq T\right\}$, the calculation is shown in Equations (23, 24). The sequence of $x_{t^{\prime }}$ is used to predict the destination $y_{t+1^{\prime }}$ at *t* + 1′ through LSTM-decoder. During the prediction process, we found that some destinations were highly correlated during certain periods of time. Most of the destinations of online car-hailing during the morning rush hour (7:00–9:00) are companies, schools, and hospitals. In the evening peak (16:00–19:00), there are more residential clusters. The temporal attention mechanism learns the long time dependent characteristics of historical data and assigns higher weights to more relevant destinations over a particular time period. Therefore, we can grasp the underlying movement patterns by analyzing destination weights and making the attention model easier to interpret.

(23) $$\begin{array}{l} M=V_{e}\cdot \sigma \left(\left({\left(x_{i}^{\left(r-1\right)}\right)}^{T}W_{1}^{\prime }\right)W_{2}^{\prime }\left(W_{3}^{\prime }x_{i}^{\left(r-1\right)}\right)+b_{e}\right) \end{array}$$

(24) $$\begin{array}{l} M_{i,j}^{\prime }=\frac{\exp\left(M_{i,j}\right)}{\sum_{j=1}^{T_{r-1}}\exp\left(M_{i,j}\right)} \end{array}$$

where *V*_*e*_,*b*_*e*_ $\in {\mathbb{R}}^{T_{r-1}\times T_{r-1}}$, $W_{1}^{\prime }\in {\mathbb{R}}^{N}$, $W_{2}^{\prime }\in {\mathbb{R}}^{C_{r-1}\times N}$, $W_{3}\in {\mathbb{R}}^{C_{r-1}}$ are learnable parameters. The temporal attention matrix M is determined by varying inputs. The value of an element *E*_*i,j*_ in *E* semantically represents the correlation strength between the time *i* and time *j*.

## 4. Experiments

### 4.1. Data Sets and Preprocessing

The data used in this study are the Haikou Didi Chuxing online car-hailing data set from March to December 2017. Each record contains the following fields: order ID, order type, departure longitude, departure latitude, arrival longitude, arrival latitude, departure time, arrival time, and travel distance between the starting point of the itinerary and the destination. Taking into account the regional land use, select the travel date of the administrative region's latitude interval [19.31, 20.03] and the longitude interval [110.07, 110.42] as the study area, and process the corresponding latitude and longitude interval into a 100 × 100 grid area, each grids actual size of the grid unit is 0.79 × 0.29 km^2^. We use the method in Chapter 3 to preprocess the data set, and process the grid data of the study area into two parts of data: One is a 100 × 100 adjacency matrix, which describes the spatial relationship between destinations; the other is the destination matrix, which describes the change in the number of online car-hailing destinations on each grid over time, the rows represent a destination location; the column represents the number of arrivals at the destination during that time period.

### 4.2. Baseline Methods

In order to further verify the effectiveness of the STAGCN model introduced in this article, we compare it with the following algorithms:

- HA: Historical average method, its predicted value is the average number of car-hailing cars arriving at their destination. In this article, we use the average of the last 100 time-gap destinations to predict the value of the next time-gap.
- MLP (Song et al., 2020b): Based on the use of fully connected neural networks, combined with the idea of regression to predict the end of the taxi, and achieved very good accuracy, we also use it as the prediction baseline of this article [11].
- LSTM (Gui et al., 2021): Because LSTM has more advantages in the learning of long sequences, it is natural to consider using it for the prediction task of this article, and as a comparison.
- GCGRU (Li et al., 2018):A method of combining gated recurrent unit network with graph convolutional network.
- STGCN (Yu et al., 2018): This model improves the conventional recursive module and convolution unit in the traditional model. The GCN module captures the spatial-temporal characteristics of the traffic graph and predicts the traffic flow through the time series model.
- STAGCN-NO-GCN: Remove the GCN module from the spatial-temporal attention graph convolutional network model proposed in this article.
- STAGCN-No-Attention: Remove the attention module from the spatial-temporal attention graph convolutional network model proposed in this article.

### 4.3. Experimental Setting

Parameter Settings during STAGCN model training are as follows: First, mean square error (MSE) is selected as the loss function of model training, and the segmentation strategy of the training set and test set for destination prediction modeling is as follows: the first 70% of the data set is taken as the model training set, and the remaining 30% is taken as the test set. In addition, the min-max function was used to standardize the relevant sequence data to the interval [0,1]. The basic parameter configuration of the benchmark deep learning model is the same as that of the STAGCN model. The specific configuration is as follows: the batch size is 100, the neuron discards rate of each layer is 0.3, and the learning rate parameter is 0.01. The default neural network layer number of the benchmark deep learning model is set at 2, and the number of neurons in each hidden layer is 100. Adam function is used as a model training optimizer for all deep learning models. The size of the filters parameter for graph convolution is set to 64, and the number of neurons in each layer of the LSTM network is set to 100. The activation function of the output layer of the model adopts a Linear function for the final prediction. Finally, RMSE and MAE are used as model error analysis indexes to evaluate the prediction performance of each model. The error indexes are calculated as follows:

(25) $$\begin{array}{l} LOSS=MSE=\frac{1}{n}\overset{n}{\underset{i=1}{\sum }}{\left(y_{i}-\hat{y_{i}}\right)}^{2} \end{array}$$

(26) $$\begin{array}{l} RMSE=\sqrt{\frac{1}{n}\overset{n}{\underset{i=1}{\sum }}{\left(y_{i}-\hat{y_{i}}\right)}^{2}} \end{array}$$

(27) $$\begin{array}{l} MAE=\frac{1}{n}\overset{n}{\underset{i=1}{\sum }}|y_{i}-\hat{y_{i}}| \end{array}$$

### 4.4. Experiment Results and Discussion

**Figure 4:**
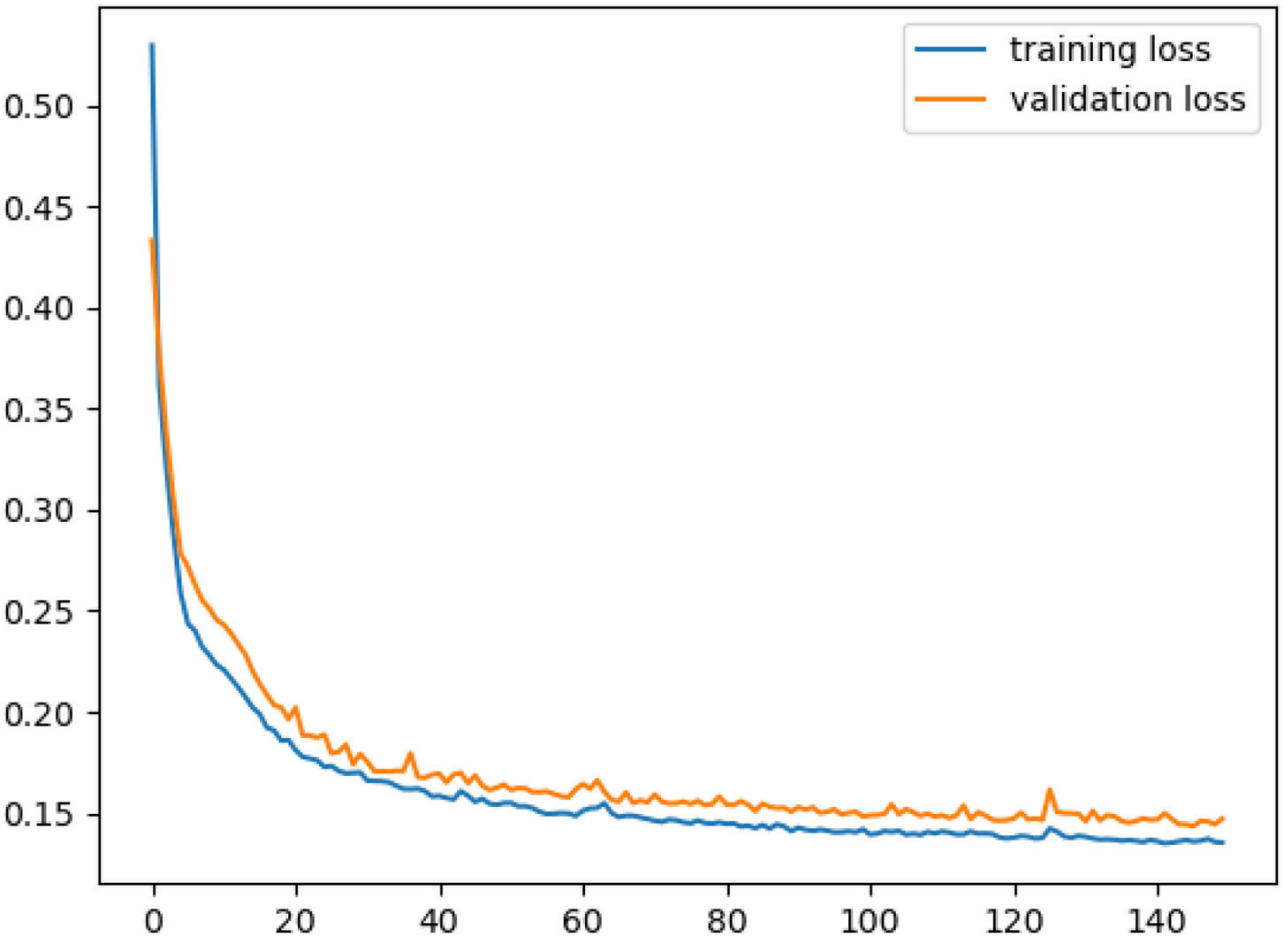
Variation for training loss and validation loss.

As shown in Figure 4, during the training process, the error gradually decreases with the increase in the number of iterations. When the number of iterations reaches a certain number, the training error rate tends to be stable and the model training is completed.

#### 4.4.1. Comparison With Baselines

**Figure 5:**
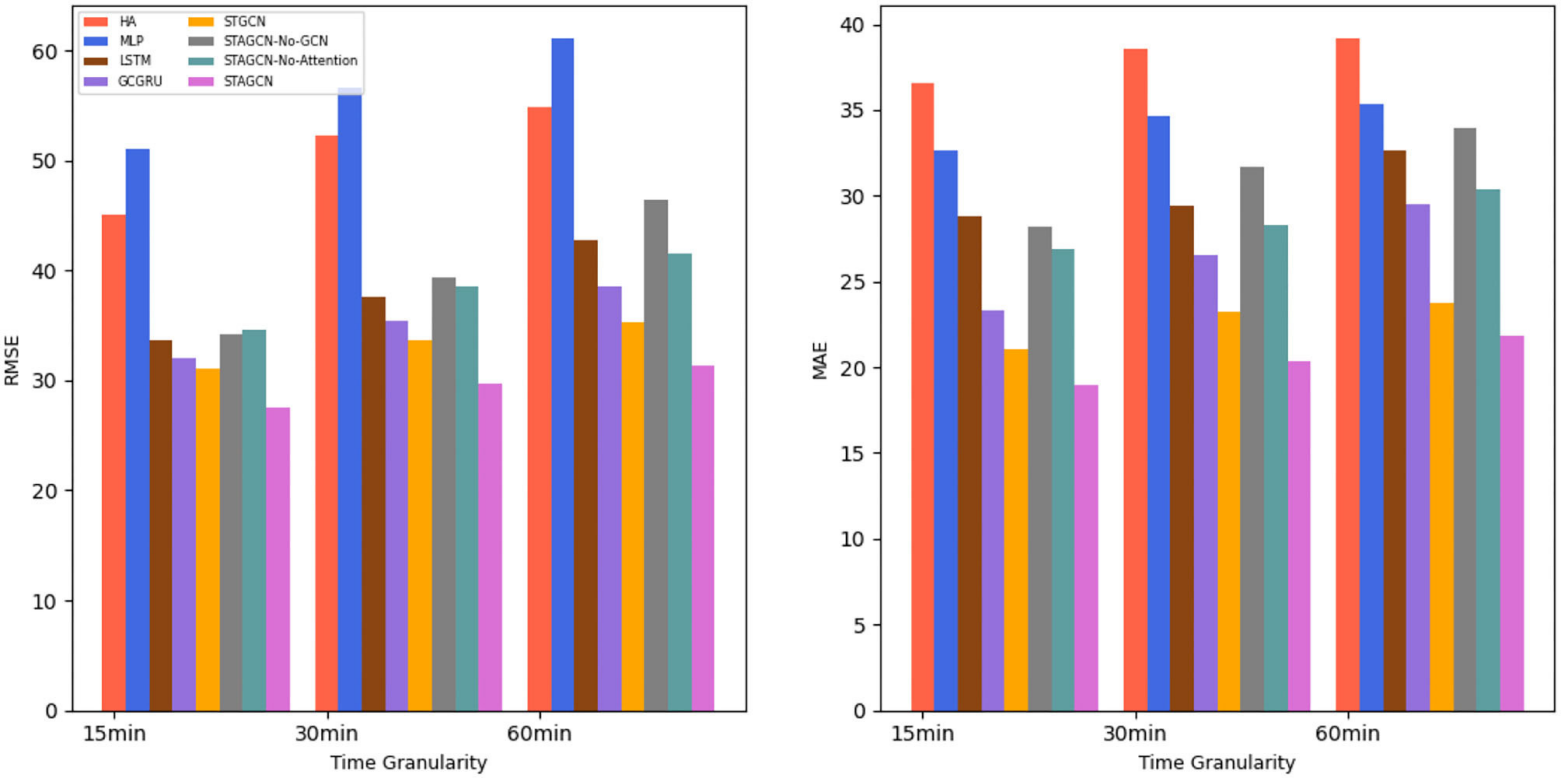
Comparison of average performance of the different methods.

Based on the real data set for experimental analysis and evaluation, the overall prediction performance of the STAGCN model and other comparative models are compared during the experiment. The experimental comparison results are shown in Figure 5 and Table 1, which show the average destination prediction error RMSE and MAE results of the STAGCN model and the comparison model at 15 min, 30 min, and 60 min. In model comparison, baseline methods are divided into three categories: traditional methods using only historical information (HA), machine learning models with temporal data as input variables (MLP, LSTM, and GCGRU), and deep learning models with spatial-temporal data as input variables (STGCN). The STAGCN performs the other models in both metrics, demonstrating the effectiveness of our model in the destination prediction task. Based on Table 1 (15 min), we summarize three conclusions as follows:

1. The STAGCN reduces the RMSE and MAE metrics by 17.53% and 17.6% at 15 min, compared with the traditional HA method. The above experiments show that the traditional method is not suitable for spatial-temporal prediction tasks.
2. The STAGCN compared with the MLP method, the RMSE and MAE metrics at 15 min are reduced by 23.54 and 13.71%; Compared with the LSTM model, STAGCN reduces the two metrics at 15 min by 6.06 and 9.83%, respectively; compared with the GCGRU model, the two metrics are reduced by 4.38 and 4.35%, respectively; the machine learning-based LSTM and GRU models have better prediction accuracy than the traditional statistical models, but only the time dependence is considered.
3. The STAGCN compared with the STGCN model, the RMSE and MAE metrics at 15 min are reduced by 3.55 and 2.08%. Although the STGCN model models spatial-temporal correlation, it does not consider the spatial-temporal characteristics of key locations.

**Table 1:** Comparison of prediction performances in different models.

**Model**	**15 min**		**30 min**		**60 min**	
	**RMSE**	**MAE**	**RMSE**	**MAE**	**RMSE**	**MAE**
HA	45.12	36.56	52.23	38.55	54.83	39.17
MLP	51.13	32.67	56.66	34.61	61.13	35.36
LSTM	33.65	28.79	37.56	29.38	42.81	32.65
GCGRU	31.97	23.31	35.41	26.56	38.53	29.35
STGCN	31.14	21.04	33.73	23.20	35.36	23.76
STAGCN-No-GCN	34.25	28.21	39.36	31.67	46.48	33.92
STAGCN-No-Attention	34.67	26.87	38.55	28.26	41.53	30.35
STAGCN(ours)	27.59	18.96	29.67	20.31	31.53	21.85

Compared with the traditional model, the STAGCN model can be applied to handle spatial-temporal data based on graph structure representation, and the spatial-temporal attention mechanism can better reveal and illustrate the potential destination and improve the performance of destination prediction.

#### 4.4.2. Ablation Analysis

We conduct further STAGCN ablation experiments. The experiments are performed by reducing the relevant modules for comparative analysis of STAGCN to measure the performance gain of different modules in STAGCN. For this purpose, two comparative versions of STAGCN were constructed:

1. Remove the graph convolution module and use LSTM for destination prediction.
2. Remove the spatial-temporal attention module to remove the focus spatial and temporal weights.

As shown in Table 1, STAGCN-No-GCN removes the GCN module, and the performance decreases, so we can find that GCN has some advantages in processing spatial-temporal data. STAGCN-No-Attention removes the spatial-temporal attention module and eliminates the weights given to the focused spatial-temporal data and the performance also decreases, indicating that passengers' travel is somewhat spatial-temporal dependent and preferential. The ablation experiment illustrates that each of the sub-modules in the model has a positive effect on the improvement of the prediction performance.

## 5. Discussion

In general, as the prediction interval becomes longer, the corresponding difficulty of prediction becomes greater, so the prediction error also increases. As can be seen from Figure 5, the method that only considers time correlation can achieve good results in the short-term prediction, such as MLP and LSTM, but its prediction accuracy decreases with the increase of prediction interval. In contrast, STGCN and GCGRU have slower performance degradation than those methods. This is mainly because temporal correlations, which are more important in long-term forecasts, can be taken into account simultaneously. However, when the scale of the network increases, i.e., when there are more time series considered in the model, the prediction error of the model increases, but the STAGCN error increases slowly with the increase of the prediction interval, and the overall performance is good. The STAGCN model proposed in this article can almost always achieve the best prediction performance, especially in the long-term prediction, the difference between STAGCN and other baselines is more significant, indicating that the strategy combines the attention mechanism and graph convolution can better mine the dynamic spatial-temporal patterns of data.

**Figure 6:**
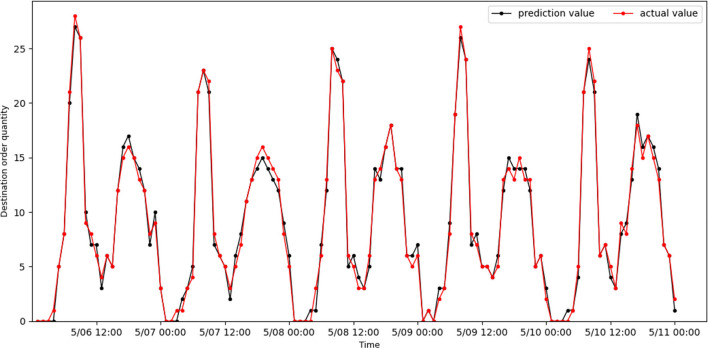
Real online car-hailing destination orders in grid 96 are compared with predicted results.

**Figure 7:**
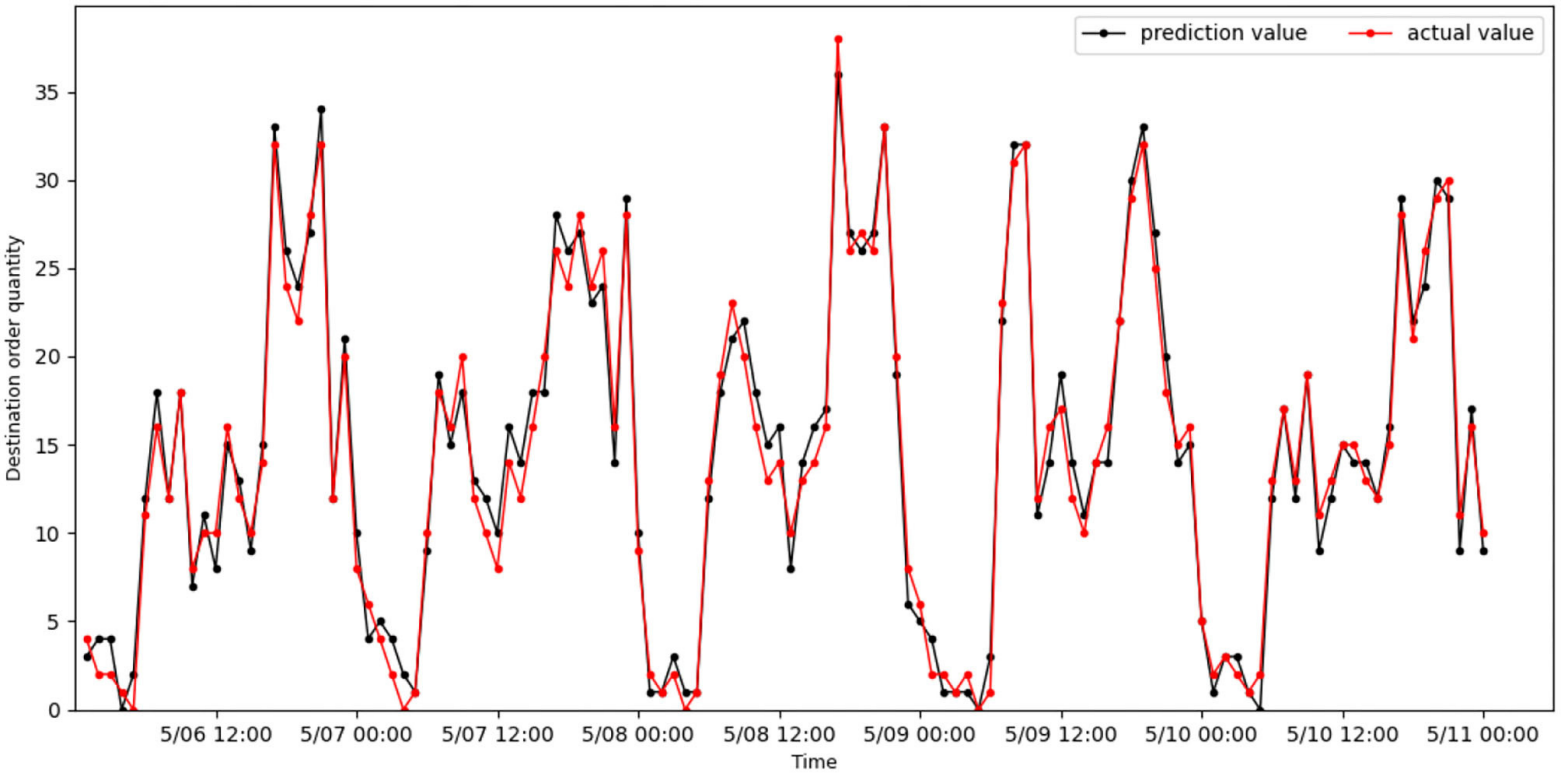
Real online car-hailing destination orders in grid 139 are compared with predicted results.

Figures 6, 7 show the actual value and predicted result of passenger destination orders through the STAGCN model (60-min interval), respectively. The predicted result shows that it is close to the real data, indicating that the model has good performance. It can be seen from the results that there are more orders in the morning peak (7:00–9:00) and evening peak (17:00–19:00), and the demand intensity of order quantity is lower in the night (such as 00:00–04:00). The No.96 grid has more office buildings and more passenger arrivals during the morning peak, while orders arriving in the area during the evening peak are less than during the morning peak. The No.139 grid has more residential complexes and fewer passenger arrivals during the morning rush hour, while more orders arrive in the area during the evening rush hour than in the morning rush hour.

In summary, through the analysis of experimental results on real data sets, the proposed spatial-temporal attention mechanism with graph convolutional network model (STAGCN) has better prediction performance than the benchmark model and the latest proposed model. Under different time gap prediction conditions, the destination prediction value of the STAGCN model can maintain a good match with the true value. At the same time, the prediction error of the model is lower than that of the benchmark model under various time gap prediction conditions. This verifies the performance of the STAGCN destination prediction model, which can effectively learn nonlinear correlation features and spatial-temporal dependence features in spatial-temporal series data.

## 6. Conclusion

Destination prediction can predict the user's destination to infer the user's intention, which in turn can make targeted recommendations. For urban transportation, it can also improve cab utilization and assist in urban transportation planning. By learning the advantages and disadvantages of existing destination prediction frameworks and methods, we address the following shortcomings: (1) The traditional statistical approach to destination prediction has a large uncertainty. It has high volatility for different data sets, high sampling process and professionalism requirements for historical data, and poor data representativeness. Traditional methods and feature construction schemes are not suitable for massive and rapidly changing historical data; (2) Historical data of destinations hide rich information about user behavior preferences, user interests, etc., and existing methods do not make full use of the spatial-temporal correlation characteristics of users; (3) Research related to destination prediction is specific to the problem of large differences between different scenarios, and many deep learning scheme models are directly applied to destination prediction. The problem of poor generality in the field of prediction makes destination prediction in the field of deep learning still have a lot of room for innovation.

This article proposes STAGCN which uses graph convolutional networks to capture spatial features, uses spatial-temporal attention mechanisms to effectively obtain dynamic spatial-temporal correlations in data and uses LSTM to generate prediction results. The STAGCN model is evaluated on a real data set. Experiments show that the proposed model achieves better performance than the five baseline methods in terms of two evaluation indicators. Compared with the traditional model, the STAGCN model is suitable for processing spatial-temporal data represented by a graph structure. The spatial-temporal attention mechanism can better reveal and explain the potential destination rules and improve the performance of destination prediction.

Destination prediction is a hot topic of current research and provides practical value for various types of location-based services. However, the field has been suffering from data sparsity and long-term dependence on historical data. In future study, to reduce the data sparsity problem and make the model applicable to predict unvisited destinations, multiple data sources, such as point of interest (POI), event datasets, and weather datasets, can be integrated to extend the model to semantic level destination prediction (Zhang et al., 2018; Jin et al., 2020). Although we use deep learning models to improve the performance of destination prediction, there are still problems such as gradient disappearance, gradient explosion, and more sensitivity to the dataset. We will investigate more advanced deep learning models and further investigate how to convolve in spatial and temporal dimensions to capture short- and long-term spatial dependencies while maintaining interpretability.

## Data Availability Statement

The datasets presented in this study can be found in online repositories. The names of the repository/repositories and accession number(s) can be found in the article/supplementary material.

## Author Contributions

CL contributed to the conception of the study and wrote the manuscript. HZ contributed significantly to the analysis and manuscript preparation. YW performed the experiment. ZW collated the experimental data and visualized the results of the experimental data. All the authors contributed to the article and approved the submitted version.

## Funding

This research was supported by the National Natural Science Foundation of China (Grant No. 61772386), Natural Science Foundation of Hubei Province (Grant No. 2020CFB795), and Zhejiang Provincial Natural Science Foundation of China General Project (Grant No. LY18F020021).

## Conflict of Interest

The authors declare that the research was conducted in the absence of any commercial or financial relationships that could be construed as a potential conflict of interest.

## Publisher's Note

All claims expressed in this article are solely those of the authors and do not necessarily represent those of their affiliated organizations, or those of the publisher, the editors and the reviewers. Any product that may be evaluated in this article, or claim that may be made by its manufacturer, is not guaranteed or endorsed by the publisher.

## References

[B1] Alzyout M. Alsmirat M. Al-Saleh M. I. (2019). Automated ARIMA model construction for dynamic vehicle GPS location prediction, in 2019 Sixth International Conference on Internet of Things: Systems, Management and Security (IOTSMS) (Granada: IEEE), 380–386.

[B2] Besse P. C. Guillouet B. Loubes J.-M. Royer F. (2018). Destination prediction by trajectory distribution-based model. IEEE Trans. Intell. Transport. Syst. 19, 2470–2481. 10.1109/TITS.2017.2749413

[B3] Cui Z. Henrickson K. Ke R. Wang Y. (2020). Traffic graph convolutional recurrent neural network: a deep learning framework for network-scale traffic learning and forecasting. IEEE Trans. Intell. Transport. Syst. 21, 4883–4894. 10.1109/TITS.2019.2950416

[B4] Dantas Nobre Neto F. Baptista C. d,. S. Campelo C. E. C. (2018). Combining Markov model and Prediction by Partial Matching compression technique for route and destination prediction. Knowledge Based Syst. 154, 81–92. 10.1016/j.knosys.2018.05.007

[B5] Do L. N. Vu H. L. Vo B. Q. Liu Z. Phung D. (2019). An effective spatial-temporal attention based neural network for traffic flow prediction. Transport. Res. C Emerg. Technol. 108, 12–28. 10.1016/j.trc.2019.09.008

[B6] Faroqi H. Mesbah M. Kim J. (2017). Spatial-temporal similarity correlation between public transit passengers using smart card data. J. Adv. Transport. 2017, 1–14. 10.1155/2017/1318945

[B7] Fu T.-Y. Lee W.-C. (2020). Trembr: exploring road networks for trajectory representation learning. ACM Trans. Intell. Syst. Technol. 11, 1–25. 10.1145/336174134336374

[B8] Gambs S. Killijian M.-O. del Prado Cortez M. N. (2012). Next place prediction using mobility Markov chains, in Proceedings of the First Workshop on Measurement, Privacy, and Mobility-MPM '12 (Bern: ACM Press), 1–6.

[B9] Gui Z. Sun Y. Yang L. Peng D. Li F. Wu H.. (2021). LSI-LSTM: An attention-aware LSTM for real-time driving destination prediction by considering location semantics and location importance of trajectory points. Neurocomputing 440, 72–88. 10.1016/j.neucom.2021.01.067

[B10] Guo K. Hu Y. Qian Z. Liu H. Zhang K. Sun Y.. (2021). Optimized graph convolution recurrent neural network for traffic prediction. IEEE Trans. Intell. Transport. Syst. 22, 1138–1149. 10.1109/TITS.2019.2963722

[B11] Guo S. Lin Y. Feng N. Song C. Wan H. (2019). Attention based spatial-temporal graph convolutional networks for traffic flow forecasting. Proc. AAAI Conf. Artif. Intell. 33, 922–929. 10.1609/aaai.v33i01.330192235155353

[B12] Hu J. Cai S. Huang T. Qin X. Gao Z. Chen L.. (2021). Vehicle travel destination prediction method based on multi-source data. Automot. Innov. 4, 315–327. 10.1007/s42154-021-00136-2

[B13] Jin G. Cui Y. Zeng L. Tang H. Feng Y. Huang J. (2020). Urban ride-hailing demand prediction with multiple spatio-temporal information fusion network. Transport. Res. C Emerg. Technol. 117, 102665. 10.1016/j.trc.2020.102665

[B14] Kan Z. Tang L. Kwan M.-P. Ren C. Liu D. Li Q. (2019). Traffic congestion analysis at the turn level using Taxis' GPS trajectory data. Comput. Environ. Urban Syst. 74, 229–243. 10.1016/j.compenvurbsys.2018.11.007

[B15] Kipf T. N. Welling M. (2017). Semi-supervised classification with graph convolutional networks. arXiv:1609.02907 [cs, stat]. 10.48550/arXiv.1609.02907

[B16] Lassoued Y. Monteil J. Gu Y. Russo G. Shorten R. Mevissen M. (2017). A hidden Markov model for route and destination prediction, I|n 2017 IEEE 20th International Conference on Intelligent Transportation Systems (ITSC) (Yokohama: IEEE), 1–6.

[B17] Li F. Gui Z. Zhang Z. Peng D. Tian S. Yuan K.. (2020). A hierarchical temporal attention-based LSTM encoder-decoder model for individual mobility prediction. Neurocomputing 403, 153–166. 10.1016/j.neucom.2020.03.080 32501365 PMC7252178

[B18] Li H. Xu X. Li X. Ma S. Zhang H. (2021a). Characterizing the urban spatial structure using taxi trip big data and implications for urban planning. Front. Earth Sci. 15, 70–80. 10.1007/s11707-020-0844-y

[B19] Li W. Wang X. Zhang Y. Wu Q. (2021b). Traffic flow prediction over muti-sensor data correlation with graph convolution network. Neurocomputing 427, 50–63. 10.1016/j.neucom.2020.11.032

[B20] Li Y. Yu R. Shahabi C. Liu Y. (2018). Diffusion convolutional recurrent neural network: data-driven traffic forecasting. arXiv:1707.01926 [cs, stat]. 10.48550/arXiv.1707.01926

[B21] Liu L. Qiu Z. Li G. Wang Q. Ouyang W. Lin L. (2019). Contextualized spatial-temporal network for taxi origin-destination demand prediction. IEEE Trans. Intell. Transport. Syst. 20, 3875–3887. 10.1109/TITS.2019.2915525

[B22] Liu X. Gong L. Gong Y. Liu Y. (2015). Revealing travel patterns and city structure with taxi trip data. J. Transport Geogr. 43, 78–90. 10.1016/j.jtrangeo.2015.01.01634752503

[B23] Lv J. Sun Q. Li Q. Moreira-Matias L. (2020). Multi-scale and multi-scope convolutional neural networks for destination prediction of trajectories. IEEE Trans. Intell. Transport. Syst. 21, 3184–3195. 10.1109/TITS.2019.2924903

[B24] Ma C. Hu X. Liu S. Liu L. (2021). Attention based multi-unit spatial-temporal network for traffic flow forecasting, in 2021 8th IEEE International Conference on Cyber Security and Cloud Computing (CSCloud)/2021 7th IEEE International Conference on Edge Computing and Scalable Cloud (EdgeCom) (Washington, DC: IEEE), 225–230.

[B25] Mahajan S. Abualigah L. Pandit A. K. Altalhi M. (2022). Hybrid Aquila optimizer with arithmetic optimization algorithm for global optimization tasks. Soft Comput. 26, 4863–4881. 10.1007/s00500-022-06873-8

[B26] Miao H. Fei Y. Wang S. Wang F. Wen D. (2022). Deep learning based origin-destination prediction via contextual information fusion. Multimed. Tools Appl. 81, 12029–12045. 10.1007/s11042-020-10492-6

[B27] Rezaie R. Li X. R. (2021). Destination-directed trajectory modeling, filtering, and prediction using conditionally markov sequences. IEEE Trans. Aerosp Electron. Syst. 57, 820–833. 10.1109/TAES.2020.3031836

[B28] Rossi A. Barlacchi G. Bianchini M. Lepri B. (2020). Modelling taxi drivers' behaviour for the next destination prediction. IEEE Trans. Intell. Transport. Syst. 21, 2980–2989. 10.1109/TITS.2019.2922002

[B29] Shan Z. Wang Y. Zhu Q. (2014). Feasibility study of urban road traffic state estimation based on taxi GPS data, in 17th International IEEE Conference on Intelligent Transportation Systems (ITSC) (Qingdao: IEEE), 2188–2193.

[B30] Song C. Lin Y. Guo S. Wan H. (2020a). Spatial-temporal synchronous graph convolutional networks: a new framework for spatial-temporal network data forecasting. Proc. AAAI Conf. Artif. Intell. 34, 914–921. 10.1609/aaai.v34i01.5438

[B31] Song Z. Wu K. Shao J. (2020b). Destination prediction using deep echo state network. Neurocomputing 406, 343–353. 10.1016/j.neucom.2019.09.115

[B32] Tao L. Gu Y. Lu W. Rui X. Zhou T. Ding Y. (2020). An attention-based approach for traffic conditions forecasting considering spatial-temporal features, in 2020 IEEE 5th International Conference on Intelligent Transportation Engineering (ICITE) (Beijing: IEEE), 117–122.

[B33] Wang L. Yu Z. Guo B. Ku T. Yi F. (2017). Moving destination prediction using sparse dataset: a mobility gradient descent approach. ACM Trans. Knowl. Discov. Data 11, 1–33. 10.1145/3051128

[B34] Wiest J. Hoffken M. Kresel U. Dietmayer K. (2012). Probabilistic trajectory prediction with Gaussian mixture models, in 2012 IEEE Intelligent Vehicles Symposium (Alcal de Henares: IEEE), 141–146.

[B35] Xu J. Rahmatizadeh R. Boloni L. Turgut D. (2018). Real-time prediction of taxi demand using recurrent neural networks. IEEE Trans. Intell. Transport. Syst. 19, 2572–2581. 10.1109/TITS.2017.2755684

[B36] Yu B. Yin H. Zhu Z. (2018). Spatio-temporal graph convolutional networks: a deep learning framework for traffic forecasting, in Proceedings of the Twenty-Seventh International Joint Conference on Artificial Intelligence (Stockholm: International Joint Conferences on Artificial Intelligence Organization), 3634–3640.

[B37] Zhang L. Zhang G. Liang Z. Ozioko E. F. (2018). Multi-features taxi destination prediction with frequency domain processing. PLoS ONE 13, e0194629. 10.1371/journal.pone.0194629 29566042 PMC5864052

[B38] Zhang X. Zhao Z. Zheng Y. Li J. (2020). Prediction of taxi destinations using a novel data embedding method and ensemble learning. IEEE Trans. Intell. Transport. Syst. 21, 68–78. 10.1109/TITS.2018.2888587

[B39] Zong F. Tian Y. He Y. Tang J. Lv J. (2019). Trip destination prediction based on multi-day GPS data. Physica A 515, 258–269. 10.1016/j.physa.2018.09.090

